# Bidirectional transcription of Linc00441 and RB1 via H3K27 modification-dependent way promotes hepatocellular carcinoma

**DOI:** 10.1038/cddis.2017.81

**Published:** 2017-03-16

**Authors:** Junwei Tang, Yu Xie, Xiaoliang Xu, Yin Yin, Runqiu Jiang, Lei Deng, Zhongming Tan, Venkatanarayana Gangarapu, Jinhai Tang, Beicheng Sun

**Affiliations:** 1 Liver Transplantation Center, The First Affiliated Hospital and State Key Laboratory of Reproductive Medicine, Nanjing Medical University, Nanjing, Jiangsu Province, China; 2 Department of General Surgery, The First Affiliated Hospital of Nanjing Medical University, 300 Guangzhou Road, Nanjing, Jiangsu Province, China

## Abstract

The retinoblastoma gene (RB1), a known tumor-suppressor gene (TSG), was decreased in multiple cancers including hepatocellular carcinoma (HCC). Here we focused on the bidirectional transcripted long noncoding RNA (Linc00441) with neighbor gene RB1 to investigate whether Linc00441 is involved in the suppression of RB1 in HCC. We found that aberrant upregulated intranuclear Linc00441 was reversely correlated with RB1 expression in human HCC samples. The gain- and loss-of-function investigation revealed that Linc00441 could promote the proliferation of HCC cells *in vitro* and *in vivo* with an apoptosis suppression and cell cycle rearrangement. Furthermore, RNA pull-down assay indicated the decreased level of RB1 induced by Linc00441 was associated with the incidental methylation by DNMT3A recruited by Linc00441. On the contrary, the transcription factor (TCF-4) enhanced H3K27 acetylation and direct transcription factor for Linc00441 was responsible for the upregulation of Linc00441 in HCC. In conclusion, the epigenetic interaction between Linc00441 and bidirectional transcripted neighbor RB1 may be a *de novo* theory cutting-point for the inactivation of RB1 in HCC and may serve as targeting site for tumor therapy in the future.

Hepatocellular carcinoma (HCC) is one of the most common life-threatening malignancies worldwide. Annually, 748 300 new liver cancer cases were diagnosed and 695 900 cancer deaths occurred worldwide. Unfortunately, nearly half of these cases and deaths were estimated to take place in China.^1, 2^ The major risk factors have been identified such as hepatitis C (HCV) and B virus (HBV) infection, toxins (aflatoxin B1), chronic alcohol abuse and nonalcoholic fatty liver disease.^3^ However, the molecular mechanisms contributing to the occurrence and development of HCC remain elusive, which may prevent the development of clinic therapeutic approaches.^4^

In recent years, more attention has been paid to investigating on long noncoding RNAs (lncRNAs).^5^ These are considered as non-protein-coding transcripts longer than 200 nucleotides and widely expressed in various organs as well as tissues of the body.^6, 7^ Depending on their proximity to the nearest protein-coding transcripts, lncRNAs can be classified into the following categories: sense, antisense, bidirectional, intronic and intergenic lncRNAs.^8, 9^ In addition, it has been reported that various carcinogenic processes, such as proliferation, apoptosis, invasion and metastasis, were closely associated with the aberrant expression of the lncRNAs.^10, 11^ LncRNA dysregulation may promote these process through modulation of chromatin remodeling, epigenetic modification, regulation of gene transcription, post-transcriptional mRNA processing, protein function and localization, and intercellular signal transduction.^12, 13^ However, the functions of most annotated lncRNAs in liver diseases remain unexplored.

The retinoblastoma gene (RB1) is the first identified tumor-suppressor gene (TSG) that is frequently inactivated in several major cancers. Researchers have revealed that the pRb protein has a key role in cell cycle regulation, particularly during the G1–S transition.^14^ When cells are stimulated by growth stimulation signals, the induced cyclin D/cdk4 can contribute to phosphorylation of RB1, which leads to the release of E2Fs from RB1-E2Fs complex and S phase entry.^15, 16, 17^ The inactivation of an upstream RB regulator (CDNKN2a/INK4a/p16) selectively occurred in these RB^+/+^ tumors resulting in almost 100% inactivation of the RB/p16 pathway in these tumors.^18^ Whether there are some other mechanisms participating in RB1 pathway inactivation is unclear.

Advancement of sequencing and annotation of whole genomes, it has been noted that bidirectional organization of coding genes is common in the whole human genomes.^19^ Among human genome, nearly 11% genes are arranged as bidirectional gene pair in the pattern of head to head, divergent manner and controlled by bidirectional promoters.^20, 21^ Majority of bidirectional promoters regulate the co-expression of a bidirectional gene pair, whereas a minority of bidirectional promoters induce the transcription initiation of one gene while inhibiting transcription in other direction.^20^ In addition, a larger part of the two genes sharing the bidirectional promoter include lncRNAs and protein-coding genes,^19^ which indicated that lncRNAs may modulate the expression of neighbor protein-coding genes in a bidirectional transcription fashion. Owing to Linc00441 and RB1 having a bidirectional promoter, we hypothesis that Linc00441 could regulate the level of RB1 protein in HCC carcinogenesis process via bidirectional pattern.

In this study, we aimed to explore the role of Linc00441 in hepatocarcinogenesis process such as cell proliferation and apoptosis. In addition, we also delineate the definite mechanisms by which the Linc00441 regulate the expression of RB1 and how epigenetic mechanisms influence the overexpression of Linc00441 in the progress of HCC.

## Results

### Aberrant upregulated intranuclear Linc00441 inversely correlated with RB1 expression

**Figure 1:**
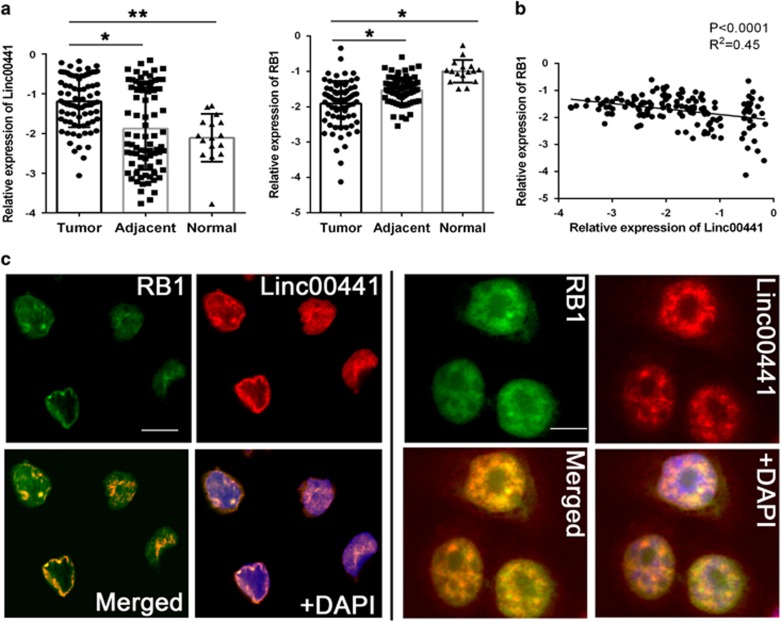
Aberrant upregulated intranuclear Linc00441 reversely corrected with RB1 expression. (**a**) Relatively expression of Linc00441 and RB1 in the tumor tissues, corresponding adjacent tissues of HCC patients (*n*=80) and healthy liver tissues (*n*=16). Data are presented as means±S.E.M. with log-transformed and analyzed with paired Student's *t*-test. (**b**) Pearson correlation analysis between the expression of Linc00441 and RB1. (**c**) Antibody-labeled RB1 (green) and probe-labeled Linc00441 (red) was detected in MHCC-97H (left panel) and HepG2 (right panel). Blue 4,6-diamidino-2-phenylindole (DAPI) staining indicates nuclei. Scale was presented at lower right of the first panel each with × 400 magnification. (*P<0.05, **P<0.01)

The bidirectional transcriptional location of Linc00441 and RB1 as predicted was presented in Supplementary Figure S1a. To investigate whether Linc00441 was involved in the pathogenesis of HCC, we first detected the expression of Linc00441 in 80 pairs HCC tumor tissues and corresponding adjacent tissues, as well as 16 healthy liver tissues. The increased level of Linc00441 was found in human HCC samples and it was proved highly associated with tumor size instead of tumor differentiation, TNM stage or tumor metastasis (Figure 1a and Supplementary Table S1). We also found that decreased level of tumor-suppressor RB1 in tumor tissues (Figure 1a). Interestingly, an inverse correlation was found in tumor tissues between Linc00441 and RB1 expression (Figure 1b) (*P*<0.001 and *R*^2^=0.45). We next analyzed the subcellular location of Linc00441 by probe hybridization in HepG2 and MHCC-97H based on the RNA expression in various cell lines listed in Supplementary Figure S1b. The probe marked Linc00441 indicated that a nuclear localization was highly consistent with the nuclear staining of RB1 (Figure 1c).

### Linc00441 promoted cell proliferation *in vitro* and associated with cell cycle

**Figure 2:**
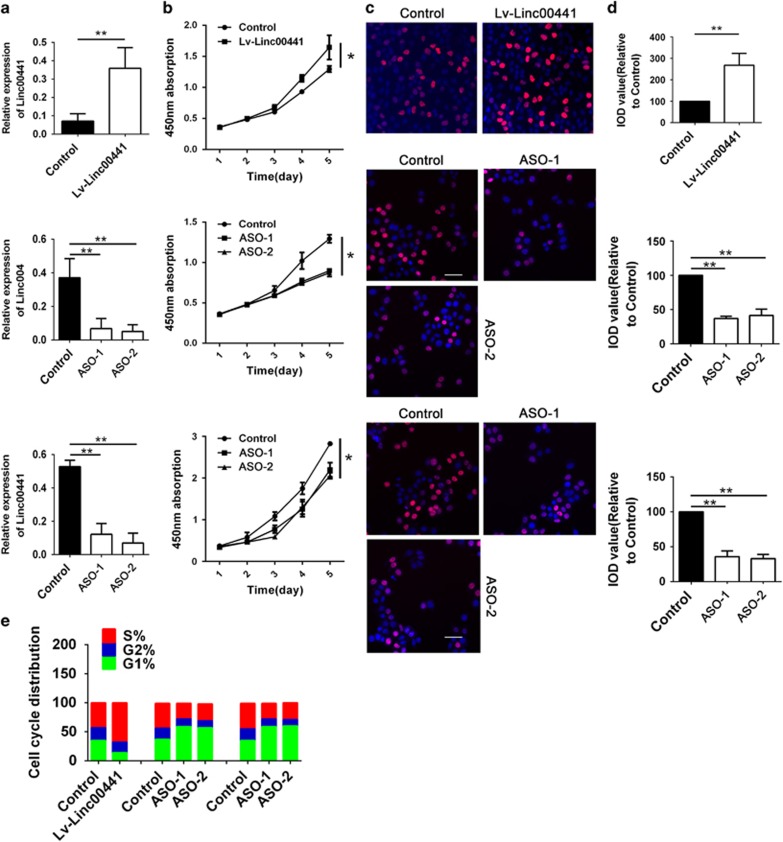
Linc00441 promoted cell proliferation *in vitro* and association with cell cycle. (**a**) Relative expression of Linc00441 in Hep3B cells treated with Linc00441 overexpression lentivirus (upper panel), MHCC-97H (middle panel) and HepG2 (lower panel) cells were treated with either ASO-1 or ASO-2. (**b**) Cell proliferation was measured by CCK8 assay kit. From days 1 to 5, each day at 450 nm absorption in different treatment group. (**c**) Cells with exponential growth was labeled with EDU (red) normalized with DAPI (blue) in different groups. (**d**) The integral optical density of cells was measured and normalized with control group. (**e**) The cell cycle distribution was listed with the percentage of G1, S and G2 phase. Data are presented as means±S.E.M. (*P<0.05, **P<0.01)

In order to investigate the possible function of Linc00441 in the development of HCC, we conducted the gain- and loss-of-function approaches *in vitro* based on the aberrant expression of Linc00441 in different HCC cell lines. After the ectopic expression of Linc00441 in Hep3B cells was confirmed (Figure 2a, upper panel), we detected the growth curve of Hep3B cells for 5 days. As presented in the upper panel of Figure 2b, we found that after culturing for 72 h, the proliferation of Hep3B was promoted by upregulation of Linc00441. Further, EDU assay at 72 h time-point by staining also confirmed the more vigorous proliferation (Figures 2c and d, upper panel). As we mentioned and confirmed the abundant expression of Linc00441 in HepG2 and MHCC-97H, the loss-of-function was applied by using ASO (antisense oligo deoxynucleotide) technology targeting Linc00441. Two independent targeting sites was designed and validated as presented in the middle and lower panel of Figure 2a. The growth curve detection by CCK8 also revealed that loss of Linc00441 could suppress the growth of both HepG2 and MHCC-97H after culturing for 120 h (Figure 2b, middle and lower panel). Further, EDU staining confirmed the repressive proliferation as presented in the middle and lower panel of Figures 2c and d.

As RB1 is frequently involved in the regulation of cell cycle, especially acting as the checkpoint of G1 phase to S phase, we next analyzed whether the loss or gain of Linc00441 can affect cell apoptosis or cell cycle. We also detected the differential distribution of cell cycle phases in the cells treated with different lentivirus mentioned above. The increased percentage of G2 and S phase was obtained in Linc00441 overexpressed cells, whereas the loss of Linc00441 could cause a G1 arrest effect (Figure 2e).

### Linc00441 enhanced tumor growth *in vivo*

**Figure 3:**
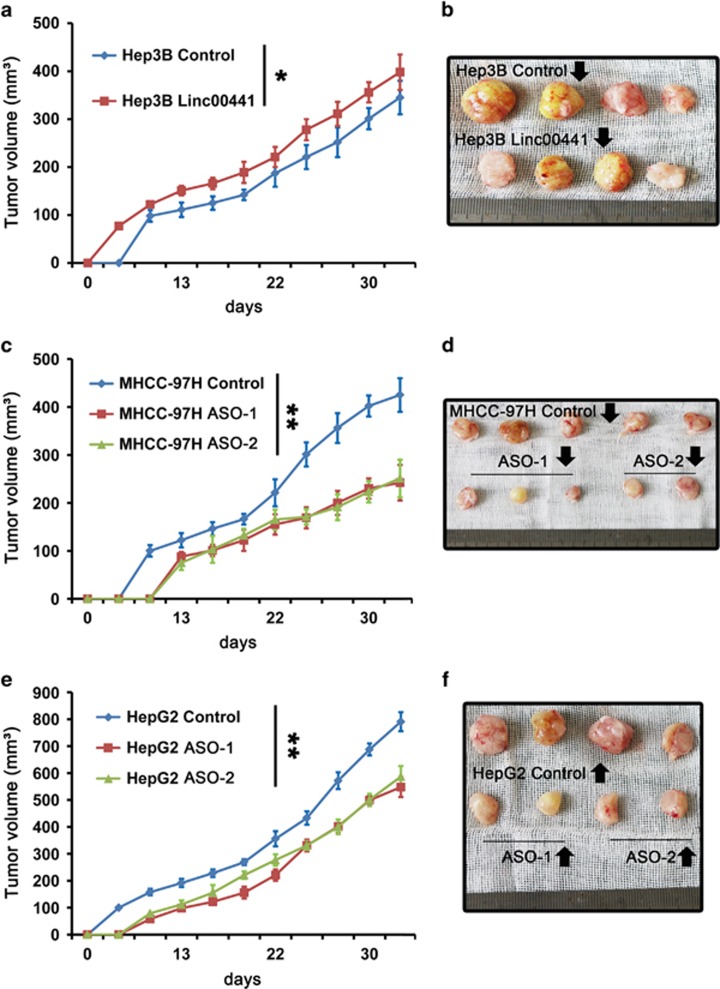
Linc00441 enhanced tumor growth *in vivo.* Mice with established tumors in different groups were measured approximately every 3 days and the tumors separated from killed mice at day 35 were presented in the right panel. (**a** and **b**) Cells treated with Linc00441 overexpression lentivirus. (**c** and **d**) Cells treated with Linc00441 ASOs and control group in MHCC-97H. (**e** and **f**) Cells treated with Linc00441 ASOs and control group in HepG2. Tumors were measured every week after the implantation, and the volume of each tumor was calculated (length × width^2^ × 0.5). Data are presented as means±S.E.M. (*P<0.05, **P<0.01)

To determine the effects of Linc00441 on tumorigenesis *in vivo*, nude mice were subcutaneously injected with Hep3B cells stably overexpressing Linc00441 and HepG2, as well as MHCC-97H cells treated with Linc00441 ASOs in a xenotransplantation model. The tumor size was monitored for about 3 days after administration. Tumor growth was markedly promoted by the upregulated expression of Linc00441 from day 5 to till killing (Figure 3a). However, this effect was strongly attenuated by the knockdown of Linc00441 with either ASO-1 or ASO-2 (Figures 3c and e). The representative segregated tumor samples were listed in the each of the right panel (Figures 3b, d and f), whereas the tumor *in situ* of each group was presented in Supplementary Figures S2a–c.

### Linc00441 induced a downregulation of RB1

**Figure 4:**
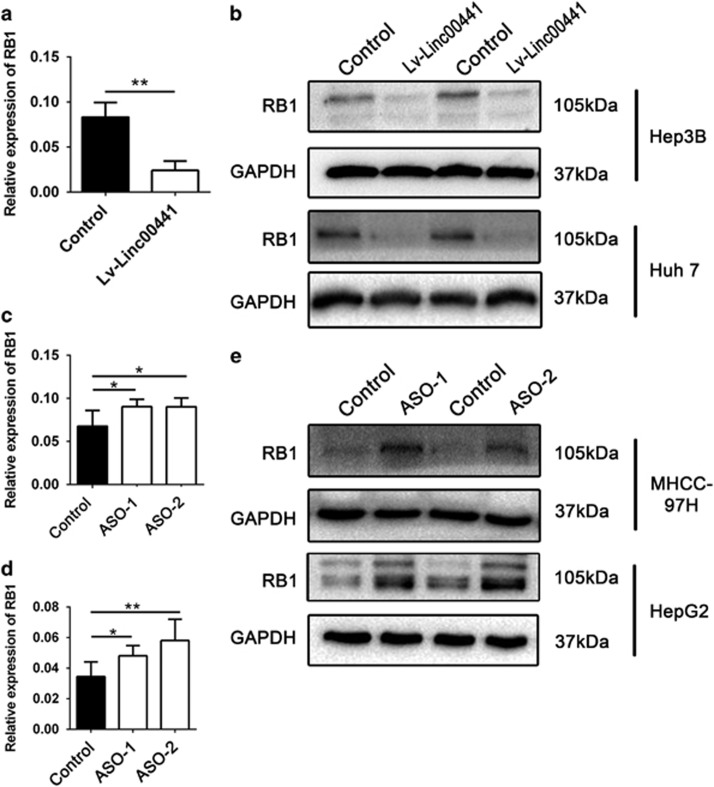
Linc00441 could suppress the expression of RB1 in mRNA and protein level. The relative expression of RB1 in Hep3B cells treated with Linc00441 overexpression lentivirus. (**b**) The expression of RB1 in protein level in Hep3B and Huh 7 cells treated with Linc00441 overexpression lentivirus. (**c** and **d**) The relative expression of RB1 in MHCC-97H and HepG2 cells treated with Linc00441 ASOs, respectively. (**e**) The expression of RB1 in protein level in MHCC-97H and HepG2 cells treated with Linc00441 ASOs, respectively

The remarkable oncogeneic function of Linc00441 from *in vitro* and in *vivo* findings promoted us to investigate whether the function of Linc00441 was associated with the neighbor gene RB1. We first detected the mRNA expression of RB1 in cells treated with either Linc00441 overexpression lentivirus or ASOs targeting Linc00441. We found that overexpressed Linc00441 cells have shown decreased expression of RB1 in Hep3B and Huh 7 cells (Figures 4a and b), whereas downregulated Linc00441 cells have shown upregulated RB1 expression in HepG2 and 97H cells in both mRNA and protein level (Figures 4c–e). Besides, according to the different abundance of RB pathway indicated by Sayan *et al.*,^22^ we detected the expression of E2F1 targets (p73 and cyclin E) to investigate whether the RB pathway was concerned. As presented in Supplementary Figure 3, both the p73 and cyclin E could be activated by overexpression of Linc00441 while suppressing by knocking down Linc00441.

**Figure 5:**
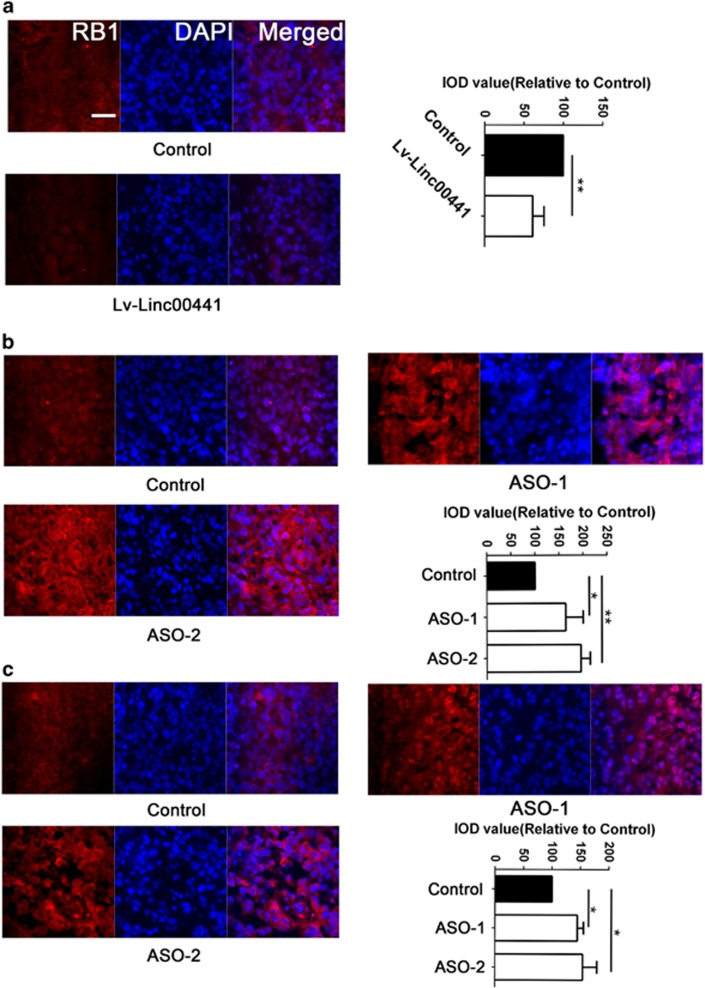
Linc00441 suppressed the expression of RB1. The expression of RB1 in Hep3B, MHCC-97H and HepG2 cells treated with Linc00441 overexpression lentivirus or Linc00441 ASOs, respectively. Frozen sections obtained from the xenotransplantation tumor were used with RB1 staining (red) merged with DAPI (blue). (**a**) Hep3B; (**b**) MHCC-97H; (**c**) HepG2. Scale was presented at lower right of the first panel with × 200 magnification. Data are presented as means±S.E.M. (*P<0.05, **P<0.01)

Frozen sections obtained from the xenotransplantation tumor mentioned above were also used with RB1 staining. We found a aberrant lower fluorescence of RB1 in the slice resected from the Linc00441 overexpressed Hep3B-induced tumor (Figure 5a). On the other hand, when we examined the optical density of RB1 stain in the section of MHCC-97H or HepG2 cells derived tumors, we found an increased optical density of RB1 normalized with DAPI when Linc00441 was knockdown by ASO technology (Figures 5b and c). Furthermore, in rescue assay, we found that knocking down of Linc00441 could not inhibit proliferation of HepG2 and 97H cells, whose RB1 gene expression was knocked down by RB1-shRNAs (Supplementary Figure 4).

### Linc00441 recruited DNMT3A by sequence special binding

**Figure 6:**
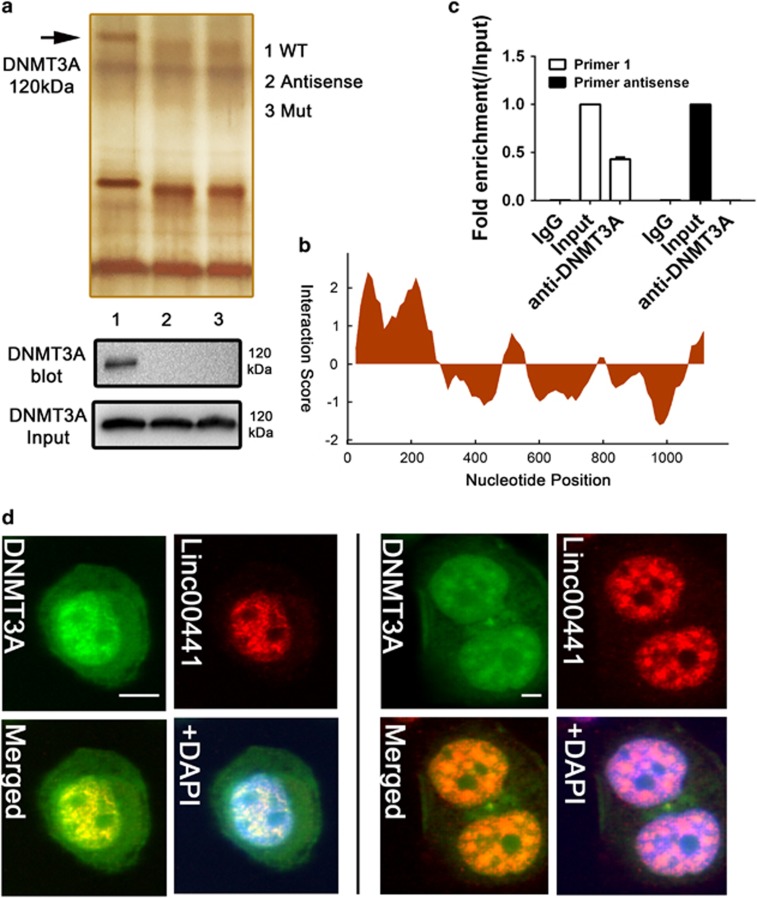
Linc00441 recruited DNMT3A by sequence special binding. (**a**) RNA pull-down assay of MHCC-97H extract from the different groups (upper panel). Specific bands were identified by immunoblotting for DNMT3A (lower panel). Line 1 indicated the wild-type (WT) full-length of Linc00441; line 2 indicated the antisense of Linc00441; Line 3 indicated the mutant type of Linc00441. The arrow indicated the band at 120 kDa approximately. (**b**) Schematic map of the potential binding site for Linc00441 in the DNMT3A protein. (**c**) RIP assay was performed using an anti-DNMT3A antibody and was confirmed with agarose gel electrophoresis using a different probe. Fold increases were calculated by comparison with the input in the lower panel. (**d**) Antibody-labeled DNMT3A (green) and probe-labeled Linc00441(red) was detected in MHCC-97H (left panel) and HepG2 (right panel). Blue 4,6-diamidino-2-phenylindole (DAPI) staining indicates nuclei. Scale was presented at lower right of the first panel each with × 400 magnification. Data are presented as means±S.E.M.

As Linc00441 could induce an inactivation of TSG RB1, we would like to unveil the detailed mechanism how Linc00441 can regulate the bidirectional transcripted neighbor gene RB1. The RNA pull-down assay was further conducted. At first, the biotin-labeled lncRNA and the antisense as control was used followed by mass spectrum validation with protein band collected at the ~120 kDa location. Through the mass spectrum screening, we found that peptide DNMT3A was most significant (Figure 6a). Based on this, we also predicted the detailed binding site of Linc00441 and DNMT3A by using the RNA sequence of Linc00441 and the amino-acid sequence of DNMT3A as input in CatRapid database. We found an enrichmental region at +20 to 300 nt of the RNA sequence of Linc00441 (Figure 6b). Further mutant type of Linc00441 was synthesized with +20 to 300 nt deletion. The RNA pull-down assay was used again and it indicated a disability of recruiting DNMT3A (Figure 6a). The results were also confirmed by western blot analysis as presented in the lower panel of Figure 6a.

RNA immunoprecipitation (RIP) by using antibody targeting DNMT3A was also conducted. We designed two independent primer of Linc00441 during the PCR assay after hybridization (primer 1 targeting the +20 to 300 nt region while primer 2 as control). By comparing with the control groups, we found that the Linc00441 could be bound by DNMT3A (Figure 6c).

Both RNA pull-down assay and RIP assay revealed that Linc00441 could recruit the DNMT3A protein. We next checked whether the location of DNMT3A was close to Linc00441. The green fluorescently labeled DNMT3A was stained in the same monoplast in both MHCC-97H and HepG2. As presented in Figure 6d, we found a colocalization of green-labeled DNMT3A and red-labeled Linc00441 in the intranuclear region.

### Enhanced H3K27 acetylation of Linc00441 promoted the methylation of RB1 through the DNMT3A-dependent approach

**Figure 7:**
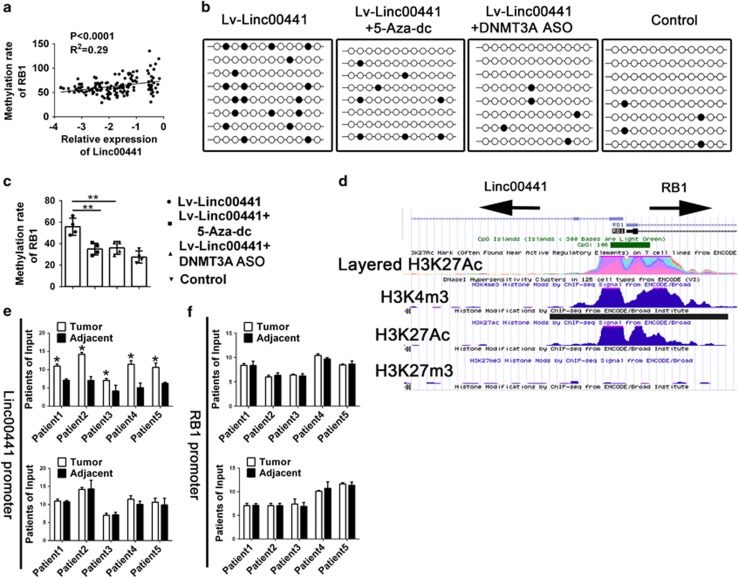
Enhanced H3K27 acetylation of Linc00441 promoted the methylation of RB1 through the DNMT3A-dependent approach. (**a**) Pearson correlation between the expression of Linc00441 and methylation of RB1 in HCC patients. The expression data of Linc00441 was log transformed. (**b**) The methylation ratio of RB1 promoter treated with different groups. Black dots indicated the methylated position. (**c**) Data statistics of methylation status in different groups. (**d**) Enrichment prediction of H3K4m3, H3K27Ac and H3K27m3 in the promoter region of Linc00441 and RB1 through ENCODE database. (**e**) The enrichment of H3K27Ac (upper panel) and H3K4m3 (lower panel) in the promoter region of Linc00441 in HCC patients. (**f**) The enrichment of H3K27Ac (upper panel) and H3K4m3 (lower panel) in the promoter region of RB1 in HCC patients.Data are presented as means±S.E.M. (*P<0.05, **P<0.01)

DNMT3A was well known as important transmitter of DNA methylation. *In vivo* expression of DNMT3A led to hypermethylation in the genome.^23, 24^ Coincidentally, the anergy of tumor-suppressor function of RB1 was also highly associated with the methylation in the promoter region in multiple human malignant tumors, including HCC.^25, 26^ After analyzing the correlation between the expression of Linc00441 and the methylation rate of RB1 in HCC samples from matched patients, we found a positive correlation between two parameters (Figure 7a). Cell treating with Linc00441 could also induce a higher methylation of RB1 in the promoter region and could be attenuated by co-treating with either 5-Aza-dc (a common inhibitor of DNMT3A) or ASO targeting DNMT3A (Figures 7b and c).

Based on the sequence analysis, we have known that Linc00441 and RB1 were located as neighbor with bidirectional transcriptional characteristic and may share the same promoter of each other. The methylation induced by DNMT3A should cause the co-suppression of both RB1 and Linc00441; however, the expression of Linc00441 was not decreased. To clarify this conflict, we analyzed the sequence feature from the epigenetical level. Interestingly, we found a remarkable 'gap' located in the attachment area especially the enrichment of H3K27Ac and H3K4m3 through the prediction by ENCODE database (Figure 7d).^27^ We thought this may be a critical point that could cause a differential expression levels of Linc00441 and RB1. We next detected the enrichment of H3K27Ac and H3K4m3 in HCC patients by ChIP assay enriching with specific antibody. As presented in Figure 7e, we found an enhanced acetylation of H3K27 in the promoter of Linc00441, whereas no difference of either H3K27Ac/H3K4m3 in RB1 promoter or H3K4m3 in Linc00441 promoter was obtained (Figure 7f).

### Transcription factor, TCF-4, promoted the transcription and H3K27Ac of Linc00441

**Figure 8:**
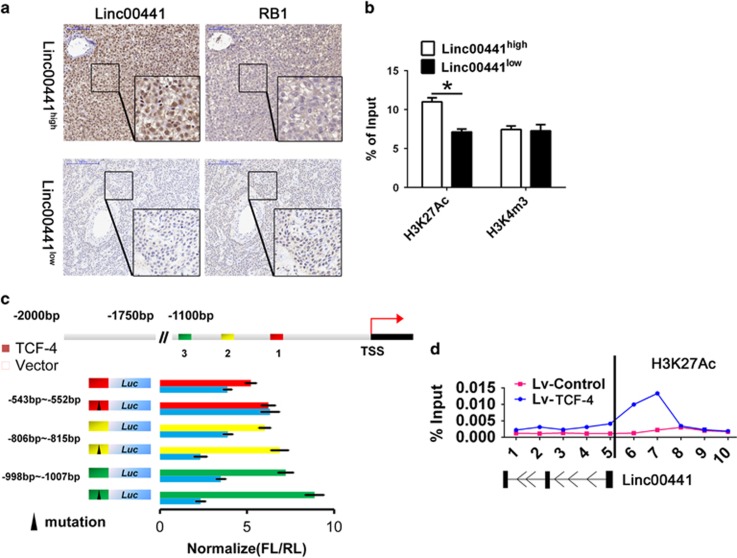
TCF-4 promoted the transcription and H3K27Ac of Linc00441. (**a**) The intensity of Linc00441 and RB1 was examined with ISH in matched sections from HCC patients in Linc00441^high^ and Linc00441^low^ group. (**b**) The enrichment of H3K27Ac and H3K4m3 from HCC patients in Linc00441^high^ and Linc00441^low^ group. (**c**) The binding sites for TCF-4 in the Linc00441 promoter regions were mutated and then cloned into the pGL4 vector as well as the wild type; the blue vector was used as the control. Cells were treated with TCF-4, and their fluorescence intensity (FL) measured by comparison with the intensity of Renilla fluorescence. The triangle indicated the mutant type. (**d**) The enrichment of H3K27Ac was detected through a multisite ChIP-PCR around Linc00441 loci in cells treating with TCF-4 overexpression lentivirus and was normalized with input. Data are presented as means±S.E.M. (*P<0.05)

The enhanced acetylation of H3K27 has been documented as an important agonist for gene expression. We next divided the HCC patients into Linc00441^high^ and Linc00441^low^ groups using the median value of Linc00441 expression as cutoff. We first detected the expression of RB1 by IHC in the matched biopsy of HCC tissues stained with Linc00441 probe by *in situ* hybridization (ISH) assay. We found that Linc00441^high^ patients displayed a lower stain of RB1, whereas Linc00441^low^ patients exhibited a higher expression of RB1 (Figure 8a). In addition, the enrichment of H3K27Ac and H3K4m3 of Linc00441 was also analyzed in these two subgroups. The enhanced acetylation of H3K27 was found in Linc00441^high^ group, whereas no difference was found regarding the enrichment of H3K4m3 (Figure 8b).

We further predicted the potential transcription factors (TFs), which mainly bind with the promoter region of Linc00441 instead of RB1 to check if there may be a special TF involved in the aberrant expression of Linc00441. As predicted, we found that TCF-4, acting as the potential TF for special transcription of Linc00441 with three potential binding sites. The dual luciferase reporter gene assay was conducted, as presented in Figure 8c, the –543 to –552 region of Linc00441 promoter was confirmed as the binding region by TCF-4 associated with the transcriptional promotion. TCF-4, a well-known TF, was involved in the promotion of H3K27 acetylation. We next analyzed the histone modifications by multisite ChIP-PCR around Linc00441 loci in cells treating with TCF-4 overexpression lentivirus. We found the H3K27 acetylation was markedly enhanced around the transcription start site of Linc00441 (Figure 8d).

## Discussion

Over the past years, many researchers have demonstrated the critical role of aberrant expression of lncRNA in occurrence and progress of several kinds of cancers including HCC.^8, 10, 28, 29, 30^ In cancer initiation and advancement, lncRNA's one of the function is epigenetic regulation.^9^ For instance, lncRNA may promote methylation of CpG island in the promoter region of a gene via recruiting DNMTs^11^ or mediates the trimethylation of H3K27 at the promoter region.^31^ Furthermore, a part of lncRNAs have been demonstrated to perform their function in the methylation process by binding to EZH2, which is the core component of polycomb repressive complex.^32, 33^

As described in the introduction, bidirectional transcription may involve in physiological function and progression of diseases. For example, Quagliata *et al.*^34^ have shown that LncRNA HOTTIP could regulate HOXA13 expression in HCC through bidirectional transcriptional pattern. Besides, Imamura *et al.*^35^ have demonstrated that LncRNA Khps1 controlled by bidirectional transcriptional manner could influence neighbor gene (Sphk1) expression through manipulating methylation pattern of the Sphk1 CpG island. Similarly, Linc00441 share a bidirectional promoter with RB1. So we explored the specific mechanisms involving Linc00441 regulation with RB1 expression. We discovered that aberrant upregulated intranuclear Linc00441 was inversely correlated with RB1 expression in human HCC samples. In *in vitro* experiments, we found that overexpression of Linc00441 could decrease the expression level of TSG RB1. In addition, analysis of the subcellular localization of Linc00441 and RB1 further confirms the expression relationship.

The retinoblastoma gene (RB1) could regulate cell proliferation in a cell cycle-dependent manner by forming RB1/E2Fs complex, which can repress the function of TF E2Fs, so high level of RB1 will increase the proportion of G_1_ phase arrest and inhibit proliferation of cells.^36, 37^ We have known that p16/p14 is responsible for the control of cyclin D/cdk4 kinase activity. Thus, in the absence of p16/p14, cyclin D/cdk4 activity is elevated, leading to Rb1 phosphorylation and E2Fs accumulation.^17, 38^ Besides, there are some studies reported that inactivation of the *p16INK4A* gene, owing to several reasons including allelic deletions of this locus and/or hypermethylation of p16/14 promoter, was found in HCCs.^39, 40, 41, 42^ In this study, we found that overexpression and decreased expression of Linc00441 may inversely regulate the G_1_ phase arrest and HCC cell lines proliferation *in vitro* and *in vivo*. Besides, we also discovered that Linc00441 can also enhance the ability of HCC cells to resist apoptosis. Taken together, Linc00441 could regulate RB1 expression, cell cycle proliferation and antiapoptotic activity.

Loss or inactivation of the RB1 genes have been identified in the majority of human tumors (*>*70%), encompassing a wide range of tumor types.^17, 43, 44^ Various kinds of mechanisms have been identified for loss or inactivation of RB1 gene expression in different types of cancers. For instance, bialleic loss of RB1 and mutations has been recognized in retinoblastoma lesions and lung cancers.^45, 46^ However, in prostate carcinoma, RB1 gene is inactivated by a deletion within its promoter region.^47^ Furthermore, CpG island methylation in the promoter of RB1 may result in the inactivation of this gene, leading to oncogenesis.^48^ As there are more CpG islands in bidirectional promoters than in non-bidirectional promoters. Thus, we hypothesis that lower expression of RB1 in HCC may be due to promoter hypermethylation in RB1 gene through the recruitment of DNMTs by Linc00441. In accordance, we found that increased expression of Linc00441 could enhance the methylation rate in the promoter region of RB1, which could be reversed by DNMTs antagonist 5-aza-2'-deoxycytidine and ASO-DNMT3A. Altogether, Linc00441 may promote epigenetic repression of RB1 gene via promoter hypermethylation. It is well known that DNMT3A is essential for *de novo* methylation and inactivated DNMT3A may have close relationships with cancers.^23, 24, 49^ Owing to lncRNA biological role as a scaffold to recruit proteins, which can modulate the gene nearby, afterward, we perform RNA pull-down assay, protein mass spectrometry RIP assay to interact between Linc00441 and DNMT3A. Bioinformatics analysis and subcellular colocalization analysis of Linc00441 and RB1 also confirmed the interactive relation between them.

Since Linc00441 and RB1 transcript in a bidirectional manner, why expression of them have shown a reverse tendency? To answer this query, we have found a 'gap' in the attachment area especially the enrichment of H3K27Ac and H3K4m3 according to the gene map. In addition, active promoter regions contain the histone modifications such as H3K4me3 and acetylation of H3K27 is considered to facilitate DNA unwinding and increase accessibility to TF binding.^50, 51^ Transcription factor 4 (TCF-4), also known as TCF7L2, is a member of the basic helix–loop–helix (bHLH) family of TFs, which could be involved in DNA binding via recognizing an E-box DNA-binding site and enhancing the transcription of genes.^52, 53^ Chromatin immunoprecipitation (ChIP) sequence experiments in various cells revealed that most of cells have shown the majority of TCF-4 sites colocalized with H3K27Ac.^54^ Luciferase reporter gene assay confirmed that TCF-4 can enhance the expression of Linc00441 and elevated TCF-4 is associated with H3K27 acetylation detected by Chip-PCR in the promoter region of Linc00441. Besides, researchers have shown that TCF-4 can also promote the expression of cyclin D1 and S phase entry and TCF-4 depletion can render cancer cells sensitive to apoptotic stimulation.^55, 56^ This may be a synergistic effect with Linc00441 on liver cancer initiation.

In conclusion, our study found that elevated expression of Linc00441 that has an inverse relationship with neighbor tumor suppressor RB1 in human HCC. Linc00441 may decrease RB1 expression through enhanced CpG islands methylation in the promoter of RB1 gene by DNMT3A recruitment, and afterward, causing proliferation of HCC in both *in vitro* and *in vivo*. TCF-4 and H3K27 acetylation also contributed to Linc00441 upregulation. Collectively, all findings in this study are in favor of understanding the mechanism of lncRNA function in HCC carcinogenesis and advancement, which can enable us to explore novel prognosis markers and potential targets for cancer therapy in near future.

## Materials and Methods

### Patient samples

Eighty paired fresh HCC tissues and corresponding adjacent noncancerous tissue samples and related clinical information were obtained from the patients undergoing hepatectomy at The First Affiliate Hospital of Nanjing Medical University. The study was approved by our Institutional Ethics Committee. All patients provided their written informed consent to participate in this study. The fresh tissue samples, which were confirmed by the histopathological examination, were collected in the operating room and processed immediately. Each sample was frozen and stored at liquid nitrogen. The clinical characteristics of all patients are summarized in Supplementary Table S1. All the cells lines authentication in this study has been carried out and by STR profiling. The antibodies were purchased from Abcam (Cambridge, CA, USA). The detailed catalog number: cyclin E1: (ab33911), TrpP73 (ab40658), RB1 (ab24), DNMT3A: (ab13888), GAPDH: (ab8245).

### The subcutaneous xenotransplantation model

Forty BALB/c nude male mice whose age were about 2 months were randomly divided into six groups including the negative control group, Linc00441 knockdown groups and Linc00441 overexpression group. After acclimating to local environment for 2 weeks, a concentration of 1 × 10^7^ cells/ml stably knocked down for Linc00441 expression in MHCC-97H, HepG2 and Hep3B-overexpressing Linc00441 and respective control cells (Linc00441-NC) were subcutaneously implanted into the left hind limb axillary of 10 BALB/C nude mice. Five weeks later, all mice were killed, the weight and size of the xenografts in each group was processed for further analysis. Animal work was permitted by the Nanjing Medical University animal studies committee.

### RIP and RNA pull-down assay

According to the manufacturer's instructions of Magna RIP RNA-Binding Protein Immunoprecipitation Kit (Millipore, Darmstadt, Germany), antibody for RIP assays of DNMT3A (ab13888) was used. The co-precipitated RNAs were detected by RT-PCR. Simultaneously, input controls and negative controls were performed to ensure that the detected signals were from RNAs specifically binding to DNMT3A.

### Quantitative methylation analysis

Genomic DNA was extracted from HCC cells, respectively, treated with the Linc00441 expression lentivirus, 5-aza-2'-deoxycytidine, DNMT3A knockdown and their control group cells. After adding sodium bisulfate from the EZ DNA Methylation-Gold Kit (Qiagen, Valencia, CA, USA) into the DNA samples, we tested the methylation in the promoter of RB1 using the method of bisulfite sequencing PCR.

### ISH and immunofluorescence (IF) microscopy

For the stain of Linc00441 by ISH, 97H and HepG2 cells were seeded into small culture dish with glass bottom and fixed in 4% formaldehyde for 15 min next day. Cells were permeabilized in PBS containing 0.5% Triton X-100 and 5 mM vanadyl ribonucleoside complex (10 mM) (New England Biolabs, Beverly, MA, USA), washed in PBS and rinsed once in SSC buffer. Hybridization was carried out using a digoxin-labeled (Roche, Mannheim, Germany) Linc00441 cDNA probes (0.5 μg/ml) in a moist chamber at 37 °C overnight. Linc00441 probe was ordered from the Abnova (Cambridge, CA, USA). After hybridization, cells were incubated with anti-digoxin–rhodamine mAb (Roche) and subjected to confocal microscopic imaging. For colocalization studies, after RNA-FISH, cells were again fixed for 5 min in 2% formaldehyde and subjected to IF staining using anti-RB1 or DNMT3A mAb (Abcam) and then stained with second antibody combined with green fluorescein and DAPI (Santa Cruz Biotechnology, Santa Cruz, CA, USA). Cells were then observed with a Zeiss (LSM 780) confocal laser scanning microscope (Zeiss, Oberkochen, Germany). For ISH in tissues, we choose chromogenic ISH method to show the expression level of Linc00441.

### IF detection

In order to determine the location of the RB1, DNMT3A as well as the relationship of one another, we added 0.5% Triton-100 to the frozen sections for 10 min and incubated the sections with the primary antibody respectively(1:200 dilution) overnight at 4 °C. The secondary antibody (red) was Alexa Fluor 546 goat anti-mouse IgG (H+L) and goat anti-rabbit IgG (1/500 dilution) for 1 h at 37 °C. The sections were added with the mounting medium and detected by confocal microscopy (Zeiss).

### Luciferase reporter gene assay

The promoter sequence of Linc00441 predicted to interact with TCF7L2, which was inserted into pGL4 promoter vector. A mutated sequence with the predicted target sites was also conducted. In all, 1 × 10^5^ cells were plated into 24-well plates. A Renilla luciferase vector pRL-SV40 (5 ng) was also co-transfected to normalize the differences in transfection efficiency. For luciferase reporter gene assay detection, the promoter of RB1 was cloned into pGL4 vector in HCC cells and cells were treated with TCF7L2.

### Chromatin immunoprecipitation

Chromatin immunoprecipitation was performed by using the ChIP assay kit according to the manufacturer's protocol (17-610, Millipore). Nearly, 1 × 10^7^*–*5 × 10^7^ cells were collected and cross-link the proteins to the DNA for 20–30 min with formaldehyde followed by sonication of lysate to shear DNA to an fragment size of 200–1000 bp. Fragment and pre-analyzed chromatin lysate was incubated overnight with the antibody anti-H3K27ac, anti-H3k27m3 and IgG, and protein A/G beads into the samples and incubated overnight at 4 °C. The crosslinking was reversed by incubation at 65 °C for 4 h. The DNA was recovered by phenol–chloroform extraction. The primers were used to detect the human RB1 promoter region by PCR.

### Statistical analysis

All experimental assays were performed independently in triplicate. Two-tailed Student's *t*-test was used to assess the statistical differences between groups. All statistical data were carried out using Statistical Program for Social Sciences 18.0 software (SPSS, Palo Alto, CA, USA) and presented with Graphpad prism 5.0 (GraphPad Software, La Jolla, CA, USA). *P-*value <0.05 was considered as significant.

Detailed Materials and methods are described in Supplementary Materials and methods.
