# Supplementary material for: Bidirectional transcription of Linc00441 and RB1 via H3K27 modification-dependent way promotes hepatocellular carcinoma
Source: Cell Death Dis. 2017 Mar 16;8(3):e2675–. doi: 10.1038/cddis.2017.81 (PMC5386573; doi:10.1038/cddis.2017.81)
Supplement: Supplementary Information [file cddis201781x1.pdf]

Supplementary Information for:

Bidirectional transcription of Linc00441 and RB1 via H3K27 modification dependent way promotes hepatocellular carcinoma

Junwei Tang[1,§], Yu Xie[1,§], Xiaoliang Xu[1],Yin Yin[1], Runqiu Jiang[1], Lei Deng[1], Zhongming Tan[1], Venkatanarayana Gangarapu[1], Jinhai Tang[1], Beicheng Sun[1,*]

1. Liver Transplantation Center of the First Affiliated Hospital and State Key Laboratory of Reproductive Medicine, Nanjing Medical University, Nanjing, Jiangsu Province, P.R.China.

[§]: The authors contributed equally to this work.

*Corresponding authors:

Dr. Beicheng Sun, Liver Transplantation Center, The First Affiliated Hospital of Nanjing Medical University, 300 Guangzhou Road, Nanjing, Jiangsu Province, P.R. China.

Phone: 86-25-68136746, Fax: 86-25-86560946

E-mail: sunbc@njmu.edu.cn

Contents:

1. Supplementary materials and methods

2. Supplementary figure legend

3. Supplementary Tables

1. Supplementary materials and methods

Quantitative real time polymerase chain reaction (qRT-PCR)

Total RNA from tissue samples and cultured cells was extracted using TRIzol reagent, according to the manufacturer's instructions (Invitrogen Life Technologies Co, CA, USA). Reverse transcription reactions were performed using the reverse transcription kit (Takara, Kusatsu, Japan), and real-time PCR was conducted in an Applied Biosystems 7900HT Detection System (Applied Biosystems®, CA, USA) following the manufacturer's instructions. Relative expression of all target genes was normalized to glyceraldehyde-3-phosphate dehydrogenase (GAPDH). To ensure the quality of the cDNA, all the pipettes were RNA/DNAse free.

Western blot

The total proteins were extracted from tissues and HCC cell lines using RIPA buffer containing fresh protease (PMSF) and phosphatase inhibitors (Beyotime, Nantong, China). Proteins were separated by SDS-PAGE and transferred to PVDF membranes (Millipore, Darmstadt, Germany). The membranes were blocked with BSA or skim milk, and then incubated with the primary antibody () at 4℃ overnight. The next day, the membranes were incubated with HRP-conjugated secondary antibodies and developed with Pierce™ ECL Western Blotting Substrate (Thermo Scientific™, CA, USA). The relative protein expression levels were normalized to GAPDH. All the experiments were repeated at least three times.

Immunohistochemical assay

The tissue samples were fixed in 4% paraformaldehyde at 4 ℃, and sectioned into slices. After removing the paraffin and rehydration, the sections were placed into

a pressure cooker for 5 min to restore the antigen in the nucleus by using the citrate method. H_2O_2 suppresses the endogenous peroxidase activity to reduce the background. *The samples were b*locked by normal goat serum and 5% BSA in TBS for 1 h at room temperature. The sections were incubated with primary antibody (1:400 dilution) overnight at 4 ℃, and then washed three times in PBS. After incubation with HRP-conjugated secondary antibody, sections were subjected to the DAB reaction. The sections were photographed by using a digital microscope camera (Nikon, Tokyo, Japan).

Cell proliferation assay

Cell proliferation was analyzed by using the CCK8 kit (Dojindo, Japan). HCC cells (Hep3B, HepG2 and MHCC-97H) transfected with lentivirus expressing Lv-Linc00441 and Linc00441-ASO were seeded at a density of 2,000–3,000 cells/well into 96-well plates and cultured for five days. Then, 10 μl CCK-8 was added to each well and the cells were incubated for 2 h. The absorbance of the medium was read at 450 nm.

For the EDU stain, HCC cells were seeded into 96-well plates and the Cell-Light™ EdU Apollo®643 In Vitro Imaging Kit (C10310-2, RIBOBIO) was used according to the manufacturer’s instructions. The plate was scanned using a high-content cytometer or imaging system (IN Cell Analyzer 2000).

Flow cytometry analysis of cell cycle

For cell-cycle analysis, MHCC-97H-control, MHCC-97H-Linc00441-ASO (antisense oligo deoxynucleotide), HepG2-control, HepG2-Linc00441-ASO, Hep3B-control and Hep3B-Lv-Linc00441 cells were subjected to serum starvation for cell cycle synchronization. The cells at the logarithmic growth period were harvested and

fixed in 70% ethanol overnight at −20 ℃. The cells were washed and incubated in propidium iodide (PI) (Multi Science) and analyzed by flow cytometry. All the experiments were repeated at least three times with triplicate.

Bioinformatic analysis

Enrichment prediction of H3K4m3, H3K27Ac and H3K27m3 in the promoter region of Linc00441 and RB1 according to ENCODE database (http://genome.ucsc.edu/ENCODE/).

2. Supplementary figure legend

Supplementary Figure 1. Detailed location information of Linc00441 and RB1 and the expression of Linc00441 in cell lines.

(a) The location of Linc00441 and RB1 in chromosome. (b) The relative expression of Linc00441 in different HCC cell lines. Data are presented as means ± SEM.

Supplementary Figure 2. Representative xenotransplantation tumor *in situ*.

(a) Hep3B cells treated with Linc00441 overexpression lentivirus. (b) MHCC-97H cells treated with Linc00441 ASOs. (c) HepG2 cells treated with Linc00441 ASOs.

Supplementary Figure 3. E2Fs associated targets expression after treating with Linc00441.

The E2Fs direct targets including p73, cyclin E showed comparatively higher expression in Linc00441 overexpression HCC lines (Hep3B) and have a lower expression in MHCC-97H and HepG2 cells when Linc00441 was knocked-down.

Supplementary Figure 4. The proliferation inhibition of knocking down of Linc00441 could be rescued by RB1-shRNAs.

(a-b) In MHCC-97H cell line, knocking down of Linc00441 could not inhibit proliferation of 97H cells, whose RB1 gene expression was knocked down by RB1-shRNAs. (c-d) Reduced Linc00441 could not inhibit proliferation of HepG2 cells, whose RB1 gene expression was knocked down by RB1-shRNAs.

Supplementary Table 1. The clinicopathological relevance analysis of Linc00441 expression in clinical samples.

Feature	Linc00441		*P* value
	Low	High	
**All cases**	40	40	
**Age**			0.823
<60	20	19	
≥60	20	21	
**Gender**			0.160
Male	29	23	
Female	11	17	
**Differentiation grade**			0.701
Well	19	18	
Moderate	14	12	
Poorly	7	10	
**Tumor Size(cm)**			**0.007**
≤3cm	27	15	
>3cm	13	25	
**Tumor Number**			0.823
Solitary	22	21	
Multiple	18	19	
**Tumor Capsular**			0.305
Incomplete	1	3	
Complete	39	37	
**TNM stage(I:II:III)**	22:10:8	28:9:3	0.218
**Metastasis**			0.626
Yes	11	13	
No	29	27	

Total data from 80 HCC patients were analyzed.

For expression of Linc00441, median expression level was used as the cutoff.

Data were analyzed by chi-squared test. *P* value in bold indicated statistically significant.

Supplementary Table 2. Oligonucleotide Sequences

Gene	Sequence	
Linc00441-1(PCR)	Forward	5'- CACCTCCAAGTGGGGACAAC-3'
	Reverse	5'- GCTGGGGTCTGGTCAAGTAG-3'
Linc00441-2(PCR)	Forward	5'- TTCCTCAGACGTTTCCACGG -3'
	Reverse	5'- GACACTTGCTGGCCTTTTGG -3'
Linc00441(RIP)	Forward	5'- TAAACTGGGAAACCTGGCGT-3'
	Reverse	5'- ATAGGGATGAGGCCCACAGT-3'
Linc00441(PCR-NC)	Forward	5'- ACATGAGACAGCCCCAAACC-3'
	Reverse	5'- TTGCATTCCTGTAGCCCACAA-3'
RB1	Forward	5'- CTCTCGTCAGGCTTGAGTTTG-3'
	Reverse	5'- GACATCTCATCTAGGTCAACTGC-3'
Linc00441 ASO-1	5' C*T*G*A*A*C*T*C*G*T*C*A*A*C*T*G*A*G*A*A 3'	
Linc00441 ASO-2	5' T*T*T*C*C*T*T*C*T*C*A*G*T*T*G*A*C*G*A*G 3'	
DNMT3AASO-1	5' C*C*A*G*C*A*A*T*T*T*C*A*G*G*C*C*A*A 3'	

DNMT3A ASO-1	5' C*C*A*A*G*C*G*T*G*T*A*T*G*A*T*G*A*A*A 3'	
ASO Control	5' G*C*C*C*A*T*T*C*A*T*T*T*C*C*T*T*C*C*C*G 3'	
GAPDH	Forward	5'- GGCATCTTGGGCTACACT-3'
	Reverse	5'- GCCGAGTTGGGATAGGG-3'

a

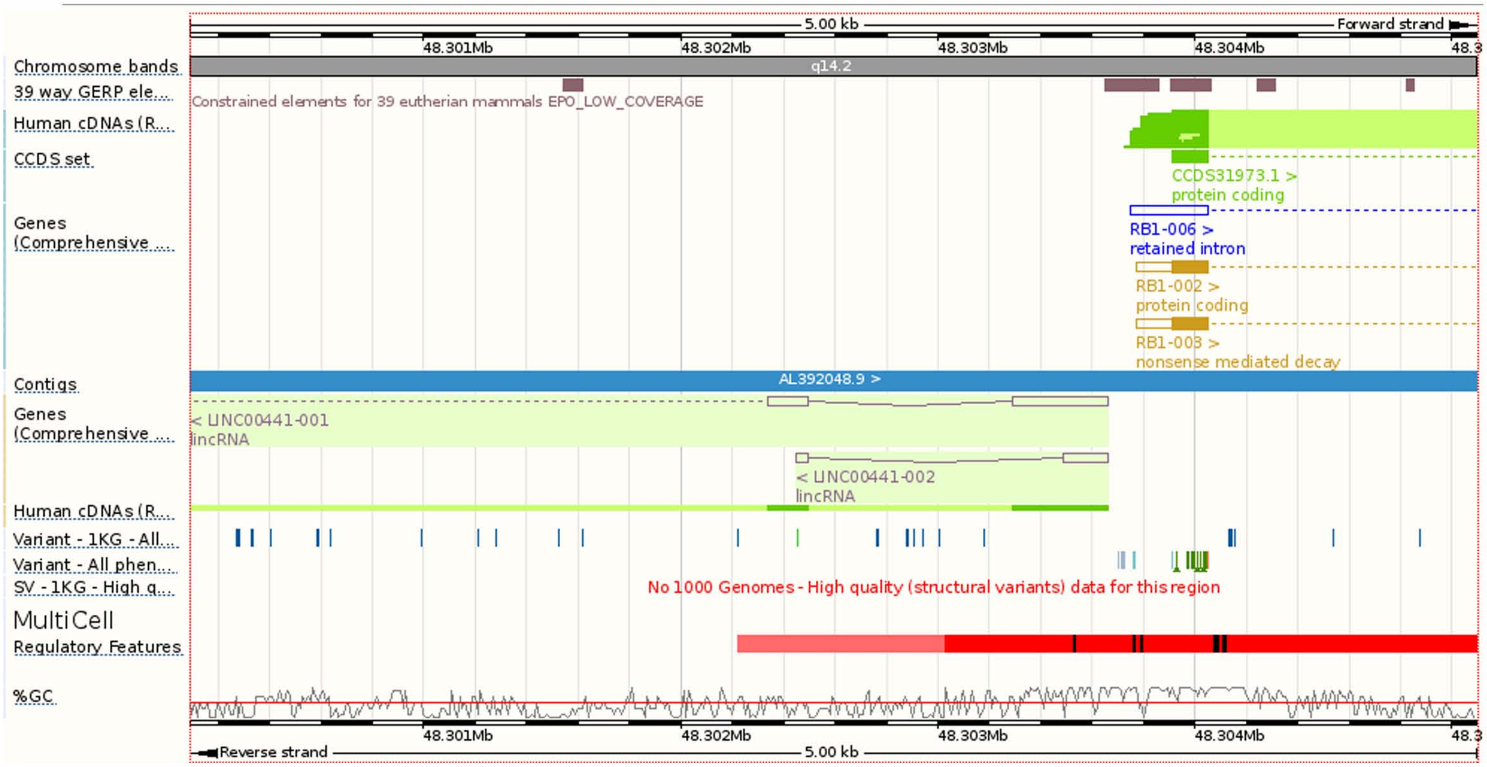
5.00 kb
Forward strand
48.301Mb
48.302Mb
48.303Mb
48.304Mb
Chromosome bands
q14.2
39 way GERP ele...
Constrained elements for 39 eutherian mammals EPO_LOW_COVERAGE
Human cDNAs (R...
CCDS set
CCDS31973.1 >
protein coding
Genes (Comprehensive ...
RB1-006 >
retained intron
RB1-002 >
protein coding
RB1-003 >
nonsense mediated decay
Contigs
AL392048.9 >
Genes (Comprehensive ...
< LINC00441-001
lincRNA
< LINC00441-002
lincRNA
Human cDNAs (R...
Variant - 1KG - All...
Variant - All phen...
SV - 1KG - High q...
No 1000 Genomes - High quality (structural variants) data for this region
MultiCell
Regulatory Features
%GC
Reverse strand

b

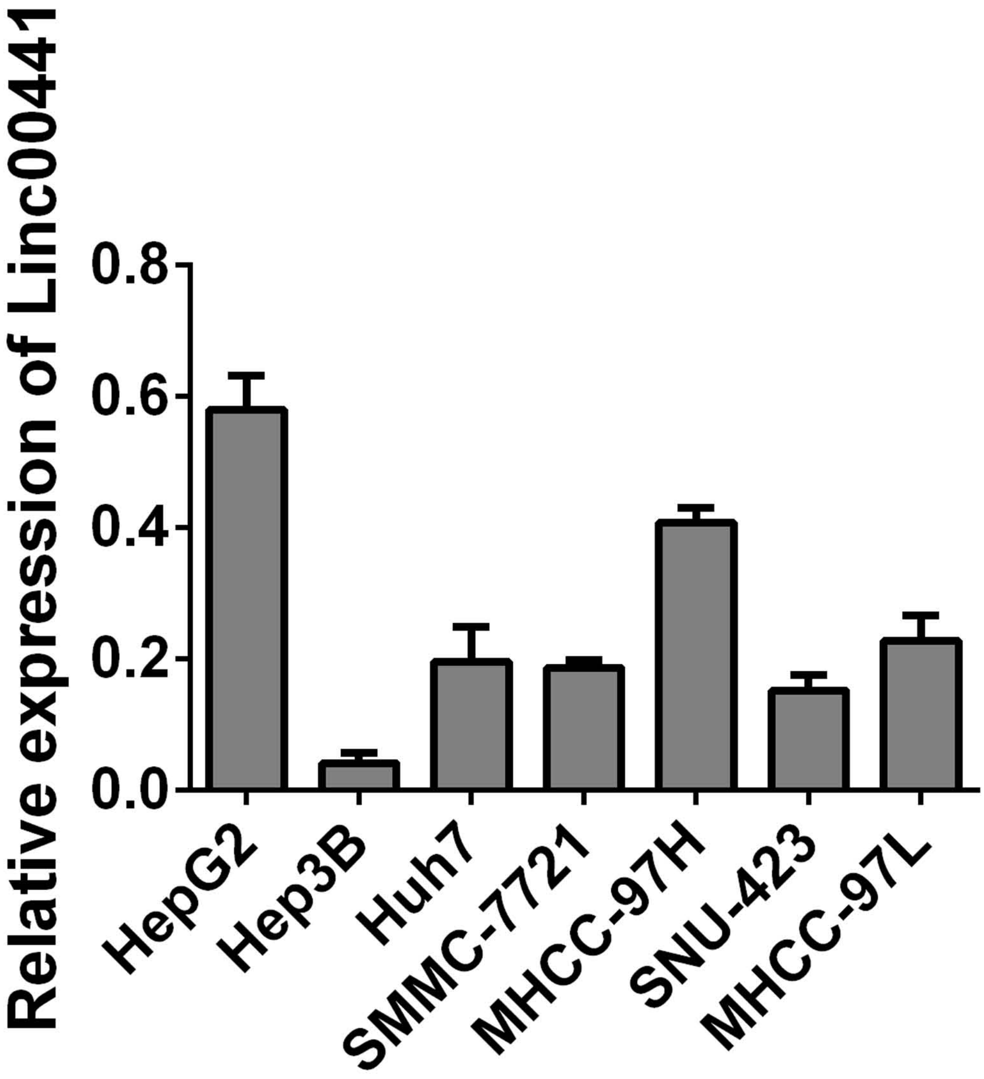
Relative expression of Linc00441
0.8
0.6
0.4
0.2
0.0
HepG2
Hep3B
Huh7
SMMC-7721
MHCC-97H
SNU-423
MHCC-97L

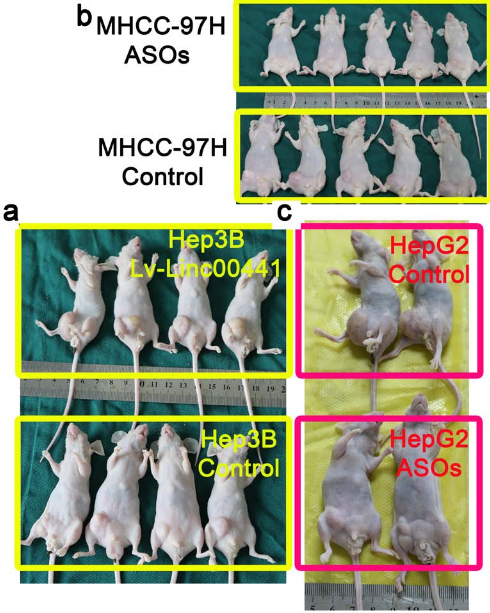

b
MHCC-97H
ASOs
MHCC-97H
Control
a
Hep3B
Lv-Linc00441
Hep3B
Control
c
HepG2
Control
HepG2
ASOs

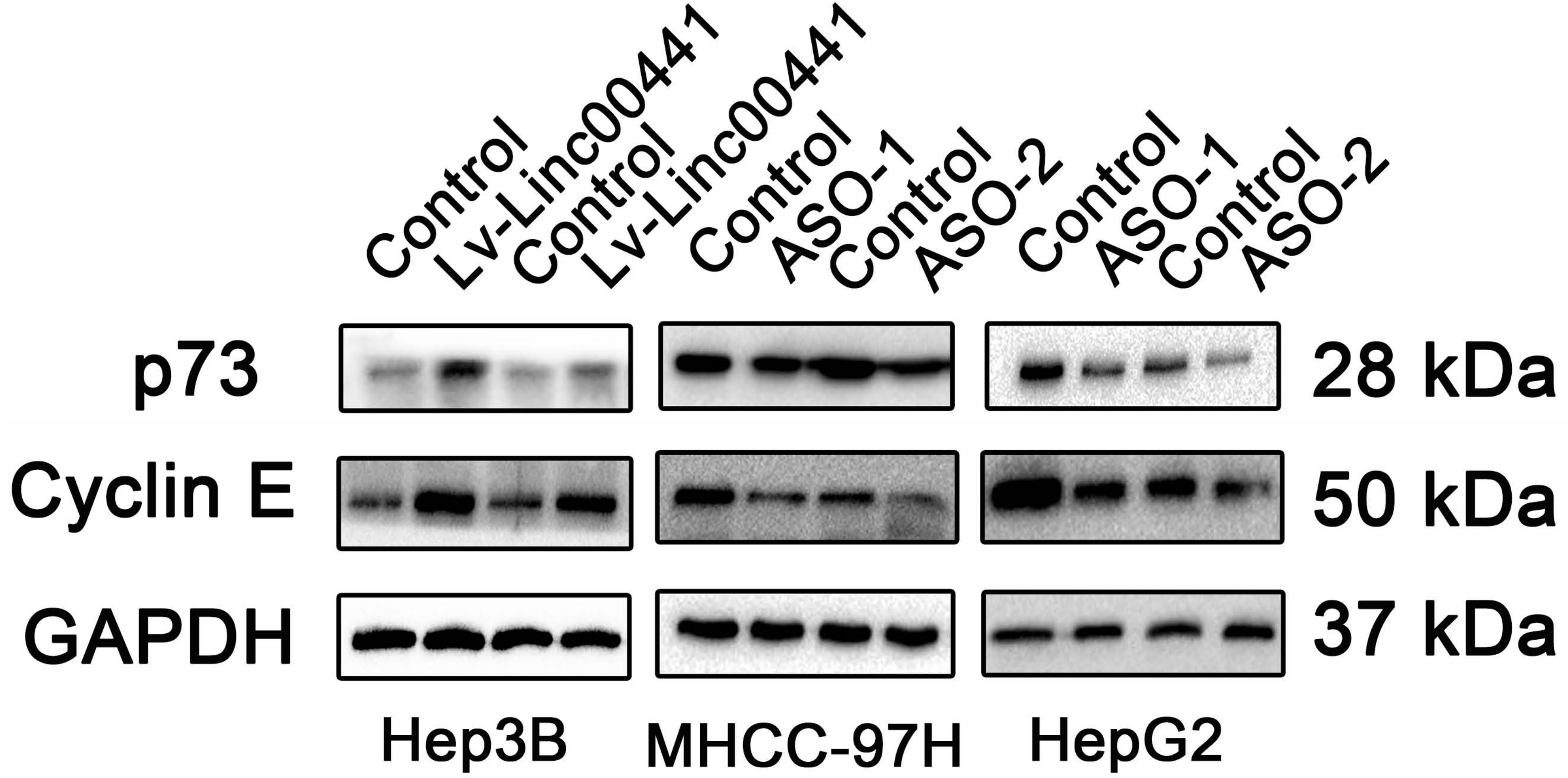
Control
Lv-Linc00441
Control
Lv-Linc00441
Control
ASO-1
Control
ASO-2
Control
ASO-1
Control
ASO-2
p73
Cyclin E
GAPDH
28 kDa
50 kDa
37 kDa
Hep3B
MHCC-97H
HepG2

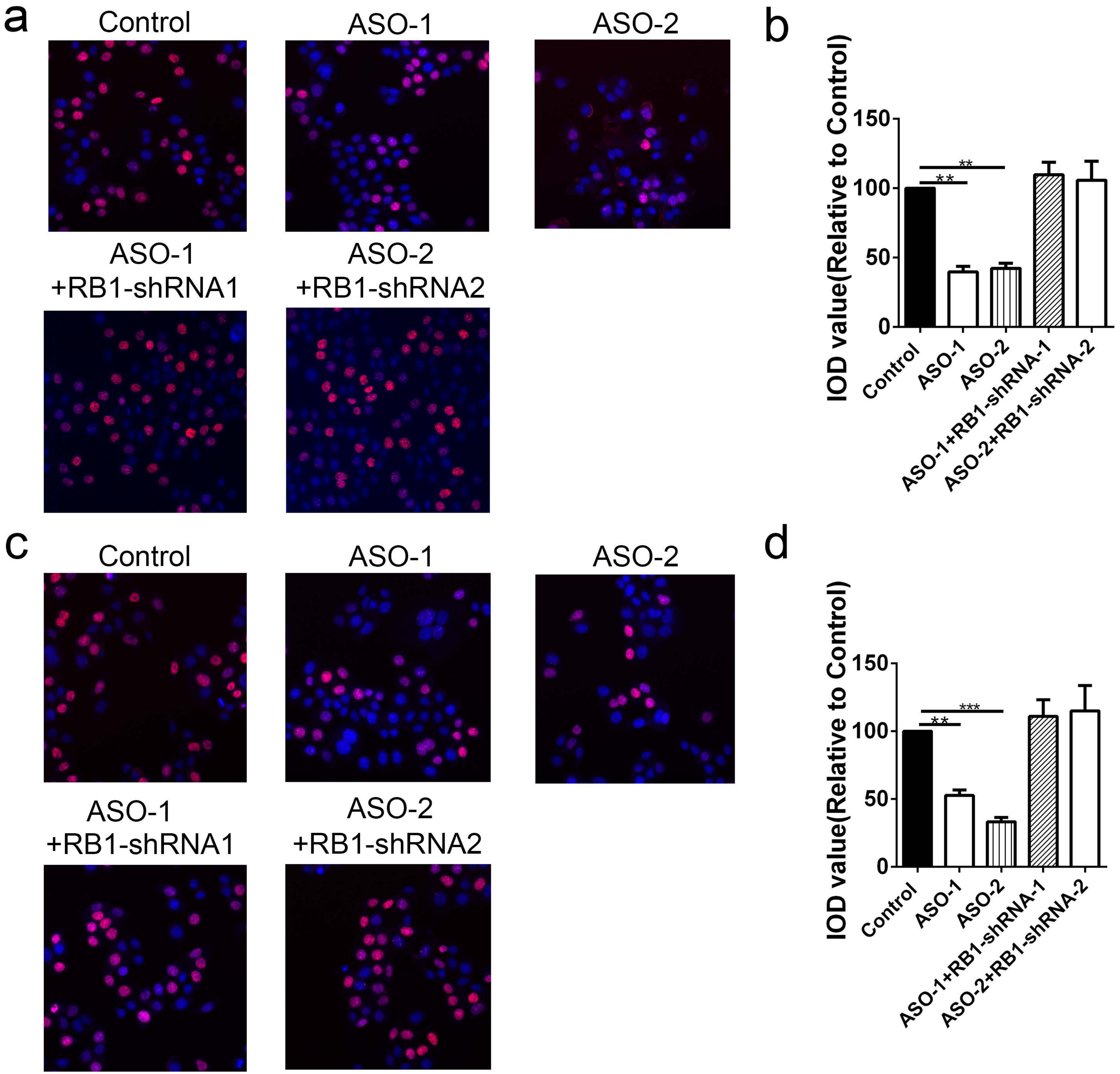

a
Control
ASO-1
ASO-2
ASO-1
+RB1-shRNA1
ASO-2
+RB1-shRNA2
b
IOD value(Relative to Control)
150
100
50
0
**
**
Control
ASO-1
ASO-2
ASO-1+RB1-shRNA-1
ASO-2+RB1-shRNA-2
c
Control
ASO-1
ASO-2
ASO-1
+RB1-shRNA1
ASO-2
+RB1-shRNA2
d
IOD value(Relative to Control)
150
100
50
0
**
***
Control
ASO-1
ASO-2
ASO-1+RB1-shRNA-1
ASO-2+RB1-shRNA-2
